# In-Depth Glycoproteomic Characterization of γ-Conglutin by High-Resolution Accurate Mass Spectrometry

**DOI:** 10.1371/journal.pone.0073906

**Published:** 2013-09-12

**Authors:** Silvia Schiarea, Lolita Arnoldi, Roberto Fanelli, Eric De Combarieu, Chiara Chiabrando

**Affiliations:** 1 Department of Environmental Health Sciences, IRCCS - Istituto di Ricerche Farmacologiche “Mario Negri”, Milano, Italy; 2 Research and Development Laboratories, INDENA S.p.A., Settala, Milan, Italy; Ghent University, Belgium

## Abstract

The molecular characterization of bioactive food components is necessary for understanding the mechanisms of their beneficial or detrimental effects on human health. This study focused on γ-conglutin, a well-known lupin seed N-glycoprotein with health-promoting properties and controversial allergenic potential. Given the importance of N-glycosylation for the functional and structural characteristics of proteins, we studied the purified protein by a mass spectrometry-based glycoproteomic approach able to identify the structure, micro-heterogeneity and attachment site of the bound N-glycan(s), and to provide extensive coverage of the protein sequence. The peptide/N-glycopeptide mixtures generated by enzymatic digestion (with or without N-deglycosylation) were analyzed by high-resolution accurate mass liquid chromatography–multi-stage mass spectrometry. The four main micro-heterogeneous variants of the single N-glycan bound to γ-conglutin were identified as Man_2_(Xyl) (Fuc) GlcNAc_2_, Man_3_(Xyl) (Fuc) GlcNAc_2_, GlcNAcMan_3_(Xyl) (Fuc) GlcNAc_2_ and GlcNAc _2_Man_3_(Xyl) (Fuc) GlcNAc_2_. These carry both core β1,2-xylose and core α1-3-fucose (well known Cross-Reactive Carbohydrate Determinants), but corresponding fucose-free variants were also identified as minor components. The N-glycan was proven to reside on Asn_131_, one of the two potential N-glycosylation sites. The extensive coverage of the γ-conglutin amino acid sequence suggested three alternative N-termini of the small subunit, that were later confirmed by direct-infusion Orbitrap mass spectrometry analysis of the intact subunit.

## Introduction

The molecular characterization of bioactive food components is essential for understanding the mechanisms of their beneficial or detrimental effects on human health.

Widely-consumed legume seeds (e.g. soybean, beans, peanut and lupin) have been studied with the specific aim of identifying and characterizing the proteins accounting for their health-promoting properties [1,2] and/or allergenic effects [3,4]. Lupin seeds, which are increasingly used in Europe as an ingredient for bakery products or as a soy substitute [4], have been characterized in relation to their interesting anti-hypercholesterolemic [5,6,7,8] and anti-hyperglycemic effects [2,9,10].

γ-Conglutin, a minor component of the mature lupin seed [2] having insulin-binding and insulin-mimetic properties *in vitro* [10,11], was found to be responsible for the anti-hyperglycemic properties of this seed [10,12]. Purified or enriched γ-conglutin lowered blood glucose in hyperglycemic rats [13], and had a substantial hypoglycemic effect in a glucose overload trial in healthy humans and rats [12]. γ-Conglutin is therefore a potential antidiabetic agent [13].

The allergenic properties of the lupin seed have been ascribed to the abundant components β- and α-conglutin [14], while for γ-conglutin the allergenic potential remains controversial, ranging from strong to weak in different *in vitro* and/or *in vivo* settings [14,15,16,17,18,19,20].

γ-Conglutin from the white lupin ( *Lupinus* *albus* ) seed is a basic 7S protein which is prevalently tetrameric or hexameric at neutral pH [2]. The monomer is composed of two disulphide-linked subunits (17k Da and 29k Da), probably deriving from post-translational proteolytic cleavage of the pro-polypeptide [2]. Proteolytic trimming of the terminal regions is a likely cause of the heterogeneity of the subunits [2,21]. The large γ-conglutin subunit reportedly carries one N-linked oligosaccharide chain [22], but the structure, the possible micro-heterogeneity, and the actual attachment site of the N-glycan have not been investigated. This subunit has two potential N-glycosylation sites, Asn_131_ within the canonical eukaryote N-glycosylation consensus sequence Asn–Xaa–Ser/Thr (where Xaa is not Pro), and Asn_132_ within the less common sequon (Asn–Xaa–Cys), recently also described in plant cells [23].

N-glycosylation is an important post-translational modification that strongly influences the structural and functional characteristics of proteins [24]. Glycoproteins have therefore been investigated in recent years to clarify the role of specific N-glycosylation features in health and disease [25,26]. Plant-specific N-glycosylation patterns are also increasingly studied in relation to the proven or potential bioactivity of glycoproteins to which humans are exposed (e.g. through food, environmental components, or nutraceutical and pharmaceutical products) [27,28]. Given the bioactivity of γ-conglutin, and considering that its (controversial) allergenic properties could potentially be influenced by the type of bound carbohydrate [28], we sought to identify the still unknown structure(s) of the N-glycan linked to the large subunit.

For the N-glycoproteomic characterization of purified γ-conglutin we used an experimental workflow based on state-of-the-art mass spectrometry [29] integrated with glycoproteomic and bioinformatic tools [30,31,32]. This approach enabled us to define the structure, attachment site, and micro-heterogeneity profile of the N-glycan bound to γ-conglutin, and provided new structural evidence that explains the heterogeneity of the protein small subunit.

## Materials and Methods

### Sample preparation

*Lupinus* *albus* γ-conglutin was kindly supplied by Professor M. Duranti (University of Milan, Italy). γ-Conglutin extracted from lupin flour was purified as described in the Supporting Information (Protocol S1).

Purified γ-conglutin (10 µg) was analyzed (n=4) under reducing or non-reducing conditions by SDS-PAGE (NuPAGE 10% Novex Bis-Tris mini gel with NuPAGE MES SDS Running Buffer, Invitrogen, Carlsbad, CA) (Figure S1).

In-gel trypsin digestion (with reduction and carbamidomethylation) was done on gel-separated γ-conglutin subunits or monomer bands according to Schiarea et al. [33]. In solution V8/trypsin digestion was done as described in detail in Protocol S1.

Dried trypsin digests of the large subunit band were treated with two N-glycosidase enzymes of different specificity, i.e. PNGase A and F [34], while dried V8/trypsin digests were deglycosylated by PNGase A only (details in Protocol S1).

### Analytical workflow

**Figure 1:**
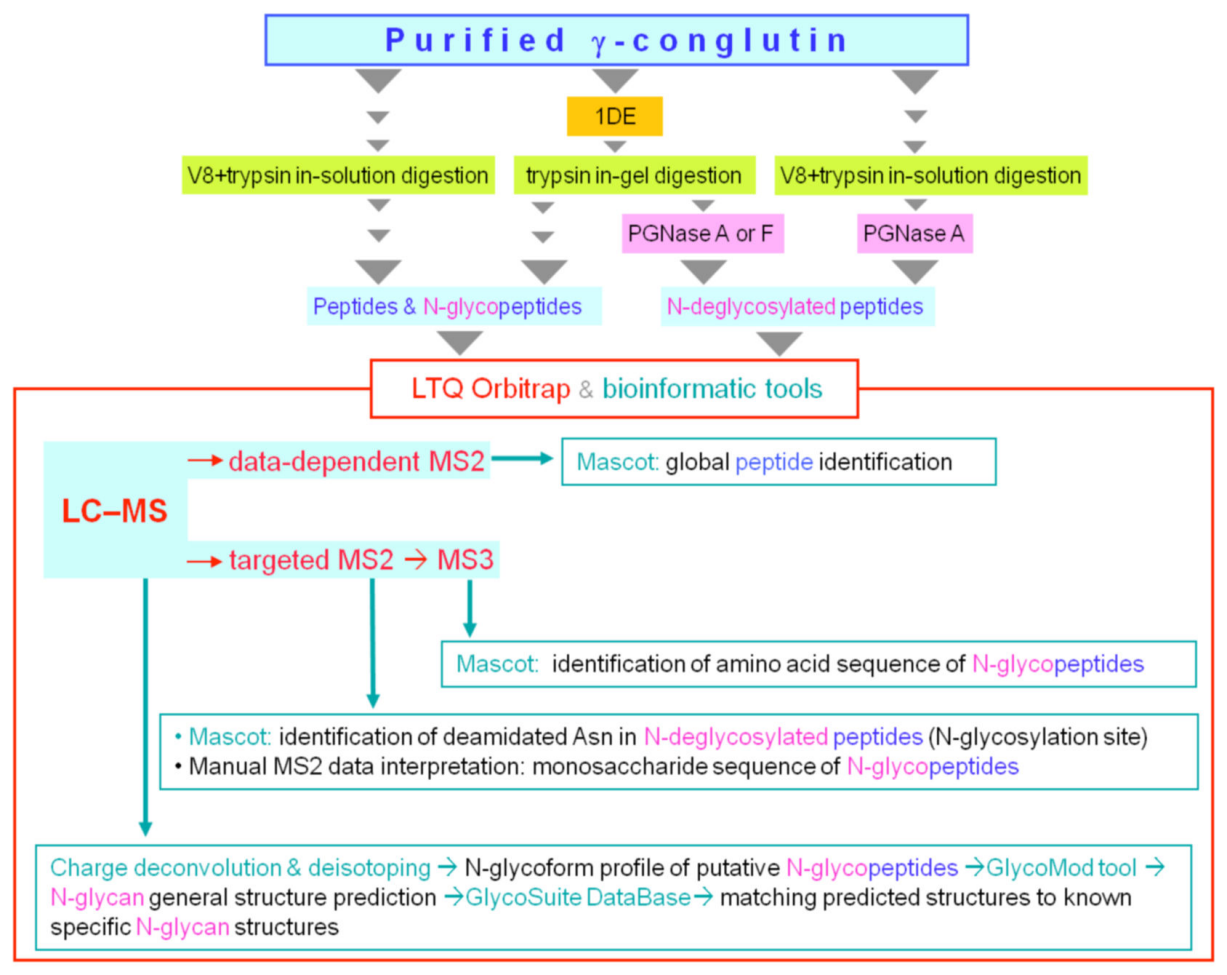
Schematic overview of the glycoproteomic workflow. Combinations of the following procedures were used: 1) reducing SDS-PAGE to isolate the N-glycosylated large subunit, 2) γ-conglutin proteolytic digestion in-gel (with trypsin) or in-solution (with endopeptidase GluC (V8) followed by trypsin) to generate different mixtures of peptides and N-glycopeptides; 3) N-deglycosylation of the digests with PNGase A or PNGase F to identify the N-glycosylation site, and assess the presence/absence of “core” α1-3 fucose. The digests (with or without N-deglycosylation) were then analyzed by LC–Orbitrap MS, with MS survey scans followed by data-dependent ITMS2 or targeted MSn. Mass spectral data analysis included the use of: 1) automated charge-deconvolution of high resolution-high mass accuracy spectra, and isotope envelope simulation; 2) bioinformatic tools for *in* *silico* glycoform structure prediction (GlycoMod and GlycoSuiteDB), and database search (Mascot) for sequence identification of non-glycosylated or enzymatically deglycosylated peptides; (3) manual inspection of MS2 and MS3 spectra of glycopeptides for sequence annotation of their monosaccharide and amino acid components, respectively.

In order to determine the structure(s), micro-heterogeneity profile, and attachment site of the N-glycan bound to γ-conglutin we used combinations of the procedures shown in Figure 1. For the in-depth sequence coverage of γ-conglutin, the non-reducing SDS–PAGE band of the protein monomer was in-gel digested with trypsin and analyzed by data-dependent LC–MS2. The heterogeneity of the intact small subunit was investigated by direct infusion–Orbitrap MS.

### Liquid chromatography–mass spectrometry (LC–MS)

The various digests were directly analyzed with an LTQ Orbitrap XL™ (Thermo Scientific, Waltham, MA) interfaced with a 1200 series capillary pump (Agilent, Santa Clara, CA, USA). Peptides/glycopeptides were separated on a C18 reverse-phase column (Thermo Scientific Biobasic 18, 150x0.18 mm ID, particle size 5µm); flow rate, 2 µl/min; eluent A, H_2_O + 0.1% formic acid; eluent B, CH_3_CN + 0.1% formic acid; gradient, 2% to 60% B in 40 min, then to 98% B in 6 min for 4 min, and re-equilibration to 2% B for 24 min. MS conditions were as follows: source DESI Omni Spray (Prosolia, Indianapolis, IN, USA) used in nanospray mode with positive ions; ion spray voltage, 2400 V; interface capillary temperature and voltage, 220°C and 42 V. The “lock mass” option was enabled for accurate mass measurements in MS mode. For CID fragmentation in multi-stage MS (MSn) mode, normalized collision energy was set at 35%. Full MS “survey” scans (m/z 400-2000) were run using the Orbitrap at resolution 60,000 at m/z 400. Each survey scan was followed by ion trap (IT) MSn analysis in “data-dependent” or “targeted” mode, as follows. For data-dependent analysis, low-resolution MS2 scans were acquired by the LTQ for the four most abundant precursor ions with isolation width 3 m/z, AGC target value of 4 x 10^4^, exclusion of singly-charged ions, and 30 s dynamic exclusion. For targeted MSn analysis, MS survey scans were followed by “targeted” MS2 scans of a pre-selected glycopeptide precursor ion. Each MS2 scan was followed by a MS3 scan of the third isotopomer of the “Peptide+HexNAc” fragment ion generated during the MS2 step.

### Data analysis

#### Automated peptide identification

The Mascot search engine (in-house version 2.2.07, Matrix Science, Boston, MA) was used to identify non-glycosylated and deglycosylated peptides in the various digests. MS2 ion search was done against the NCBInr database 20120531. All other details are shown in Protocol S1.

#### MS spectra deconvolution

Averaged high-accuracy, high-resolution MS spectra over given LC retention time-ranges were deconvoluted to obtain the singly-charged monoisotopic molecular mass of the multiply-charged glycopeptides, using Xtract for Qual Browser 2.0 (Thermo Scientific).

#### Isotope envelope simulation

The Qual Browser 2.0 Isotope Envelope Simulation tool was used to examine the correspondence between the experimental and theoretical isotope envelopes of each putative peptide glycoform (multi-charged MH^+^ ions).

#### In-silico glycoform structure prediction

GlycoMod (http://web.expasy.org/glycomod/) [35] was used to predict the structure of the tryptic peptide glycoforms. The high-resolution accurate-mass values (monoisotopic, singly-charged MH^+^) of peptide glycoforms were entered into GlycoMod together with the sequence of the γ-conglutin precursor (Q9FSH9), the digesting enzyme (trypsin), the protein chemical modification (carbamidomethylation of all cysteine residues), 3 ppm tolerance for the theoretical *vs.* experimental mass match, and no restriction on monosaccharide composition.

### Direct-infusion MS

After reduction (in 10mM DTT, 1 h at 56°C under shaking), γ-conglutin was directly infused (2 pmole/µl in MeOH/formic acid 2% (50:50, v/v) into the LTQ Orbitrap at 2 µl/min flow rate. MS conditions were: source DESI Omni Spray in nanospray mode with positive ions; ion spray voltage, 2400 V; interface capillary temperature and voltage, 225°C and 32 V. MS spectra were acquired in the Orbitrap at mass range 200-2000 m/z, resolution 100,000. Mass spectra charge-deconvolution was done with Xtract for Qual Browser 2.0.

## Results and Discussion

### N-glycoform profile by LC–Orbitrap MS

The averaged LC**–**MS spectrum of the whole in-gel trypsin digest of the large subunit was first charge-deconvoluted over the entire m/z range (LC time range: 10-40 min) to obtain a global view of the monoisotopic singly-charged pseudo-molecular ions. Considering trypsin cleavage also before proline, the molecular mass of the shortest non-glycosylated peptide encompassing the two N-glycosylation sequons (N_131_NT and N_132_TC) is 4085 Da. Above 4000 m/z the deconvoluted mass spectrum showed two main series of five peaks (series I: 4962.27–5094.31–5256.36–5459.44–5662.52 m/z; series II 6830.02–6962.07–7124.12–7327.20–7530.28) spaced by mass differences possibly due to single monosaccharide residues, i.e. 132.04 Da (pentose, Pent), 162.05 Da (hexose, Hex), 203.08 Da (N-acetyl-hexosamine, HexNAc), and again 203.08 Da. We thus hypothesized that the five peaks in series I and II represented the same five glycoforms (hereafter named A to E) of two partially overlapping tryptic peptides that encompass both potential glycosylation sites (N_131_ and N_132_), i.e. Pept_127-165_ and Pept_111-165_ (monoisotopic mass MH^+^= 4085.94 and 5953.70, respectively). The A glycoforms would thus represent Pept _127-165_ or Pept_111-165_ carrying a short glycan residue (monoisotopic mass, 876.32 Da), while B to E would have the same saccharide composition as A, with the following increasingly complex composition: B=A+Pent, C=B+Hex, D=C+HexNAc, and E=D+HexNAc. We also noted two minor glycoform series of Pept_127-165_ and Pept_111-165_ (named here B′ to E′) which are discussed below.

As expected for large peptides carrying rather small glycans, the five putative A to E glycoforms of Pept_127-165_ eluted late (around 35 min), and slightly earlier than the non-glycosylated peptide (identified by Mascot with high confidence, Figure S2). Similarly, the putative A to E glycoforms of Pept_111-165_ eluted around 34 min, but in this case the non-glycosylated peptide was not detected.

**Figure 2:**
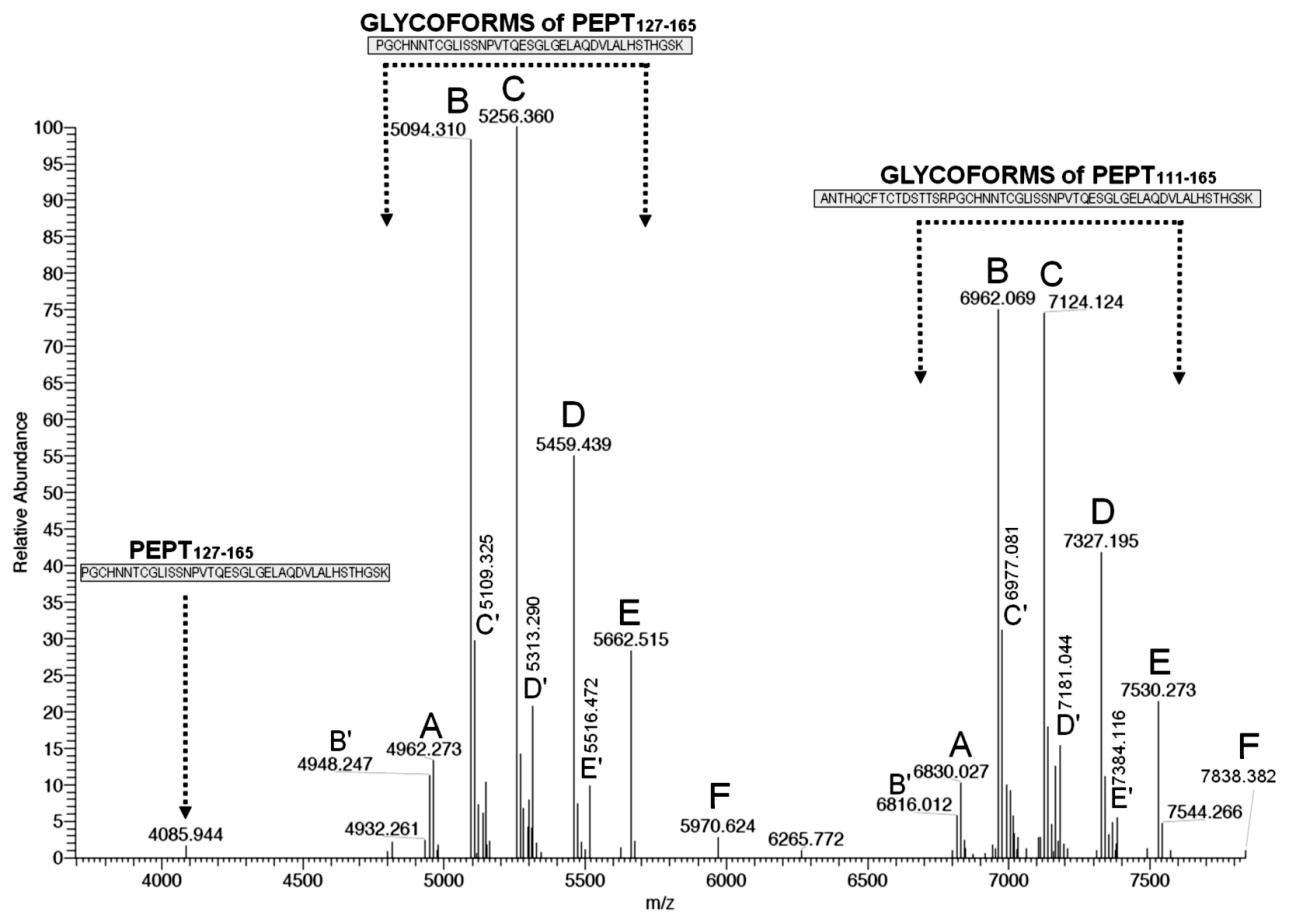
γ-Conglutin glycoform profile obtained by LC-Orbitrap MS analysis of the in-gel tryptic digest of the large subunit. The MS spectrum (averaged over the elution time of the A-E and B′-E′ glycoforms of Pept_127-165_, Pept_111-165_, and the non-glycosylated Pept_127-165_) was charge-deconvoluted and deisotoped to show the monoisotopic MH^+^ ions.

To obtain an immediate approximate view of the relative intensity of the A to E glycoforms, we deconvoluted the full MS spectrum over a time range (33.5-36 min) covering the elution of both glycoform series (as well as non-glycosylated Pept_127-165_) (Figure 2). The mass list of the deconvoluted spectrum is shown in Table S1. The relative abundance profile of the A to E glycoforms (B=C>D>E>A) was similar for all the analyzed glycopeptides (Table S2), and is presumably representative of the N-glycosylation micro-heterogeneity profile of the intact protein. The signal of the non-glycosylated Pept_127-165_ appears minimal in relation to the global intensity of the glycoforms, suggesting that even if a small proportion of γ-conglutin copies can exist without N-glycosylation the protein is mostly in the N-glycosylated form. Figure 2 also confirms the presence of minor glycoforms (B′ to E′) of Pept_127-165_ and Pept_111-165_, whose identification is discussed later. A low-abundance putative glycoform “F” was also detected for both peptides (Figure 2, m/z 5970.624 and 7838.382). The probable composition of F, showing a 308.109 Da (Hex+dHex) mass increase relative to the E glycoforms of both peptides, is therefore Hex _4_HexNAc _4_dHex _2_Pent_1_. Given the very low abundance of this glycoform, its structural analysis was not attempted.

### N-glycoform structure prediction

The experimental accurate mass values of the A to E glycoforms of Pept_127-165_ and Pept_111-165_ obtained by LC-MS were used to hypothesize the structure of the attached N-glycans by interrogating GlycoMod. A unique matching glycopeptide was obtained for each experimental mass. The predicted oligosaccharide compositions (Table 1) corresponded to N-glycan entries that are listed – with documented structural details, including complete linkage information – in GlycoSuiteDB [36], (http://glycosuitedb.expasy.org/glycosuite/glycodb).

**Table 1:** Saccharide composition predicted by GlycoMod on the basis of the experimental accurate mass of the A to E glycoforms of Pept_127-165_ and Pept_111-165_.

			**N-GlycoPept_127-165_** [M+H]^+^			**N-GlycoPept_111-165_** [M+H]^+^		
	**N-glycan composition**	**N-Glycan Residue** ^a^	**Theoretical**	**Experimental**	*Error (ppm*)	**Theoretical**	**Experimental**	*Error (ppm*)
**A**	Hex_2_HexNAc_2_dHex_1_	876.322	4962.263	4962.273	*2.0*	6830.020	6830.027	*1.0*
**B**	Hex_2_HexNAc_2_dHex_1_Pent_1_	1008.365	5094.305	5094.310	*1.0*	6962.063	6962.069	*0.9*
**C**	Hex_3_HexNAc_2_dHex_1_Pent_1_	1170.417	5256.358	5256.360	*0.4*	7124.115	7124.124	*1.3*
**D**	Hex_3_HexNAc_3_dHex_1_Pent_1_	1373.497	5459.437	5459.439	*0.4*	7327.195	7327.195	*0.0*
**E**	Hex_3_HexNAc_4_dHex_1_Pent_1_	1576.576	5662.517	5662.515	*-0.4*	7530.274	7530.273	*-0.1*

^a^ Theoretical mass

**Figure 3:**
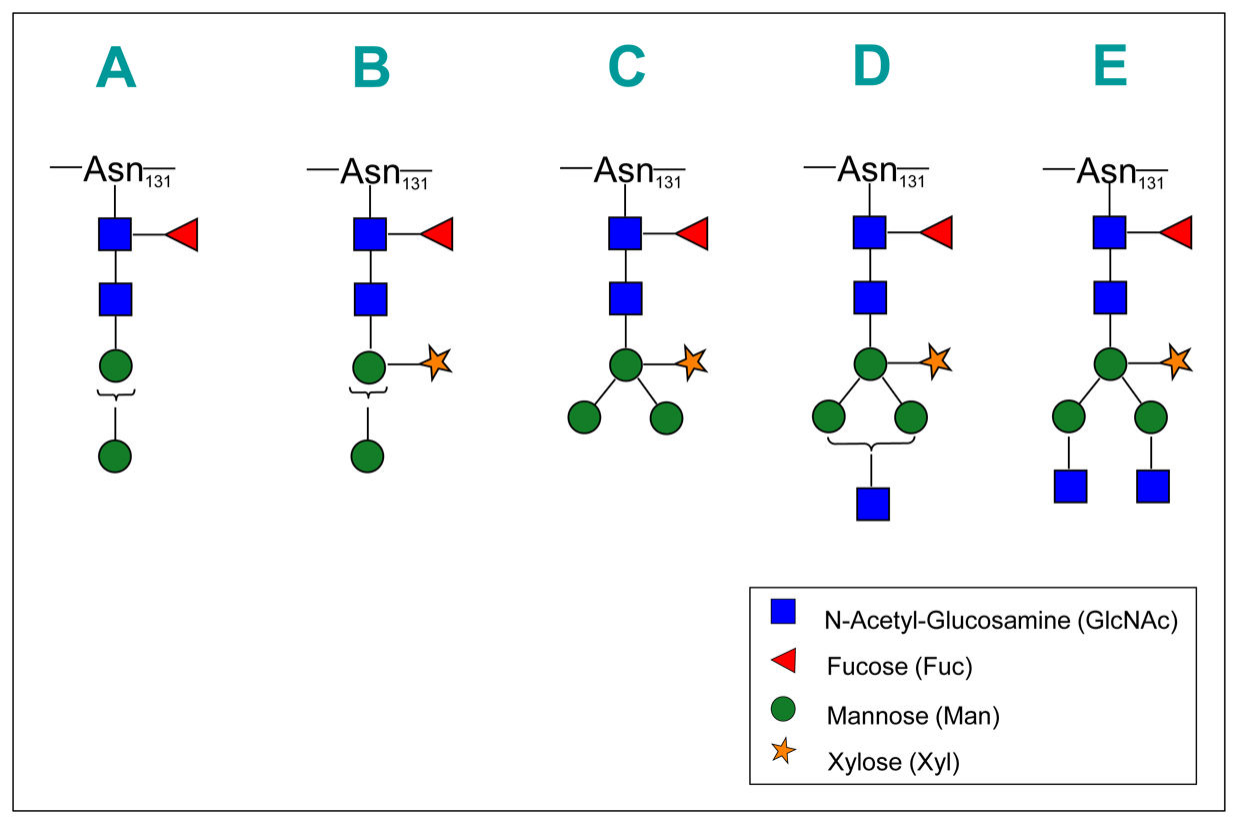
Proposed N-glycan structures for γ-conglutin glycoforms A to E. Structures are drawn using the Consortium for Functional Glycomics Symbol Nomenclature (http://www.functionalglycomics.org/static/consortium/Nomenclature.shtml), with GlcNAc=square; Fuc=triangle; Man=circle; Xyl=five-pointed star. Braces within the glycan structure indicate possible alternative linkages. For linkage details, see Table S1.

In plant organisms, the composition of our A glycoform (Hex _2_HexNAc _2_dHex_1_) matched a single specific isomeric N-glycan structure (Man_2_(Fuc) GlcNAc_2_) [37,38]. The composition of the B to E structures (Table 1) univocally matched specific N-glycans almost exclusive of plant organisms. These N-glycans have in common a core fucose residue (α1-3 linked to the terminal reducing N-acetyl-glucosamine), and a xylose residue (β1-2 linked to the bisecting mannose) (Figure 3 and Table S3). For the A, B and D structures there are two isomeric variants with an α1-3 or α1-6 arm linked to the bisecting mannose, the isomer with the α1-6 arm being most frequently (A and D structures) or exclusively (B structure) reported in GlycosuiteDB.

We confirmed the oligosaccharide composition and some sequence features of the A to EN-glycans by MSn (see below), but the *specific* structures that we propose are based on the unique-matching GlycoSuiteDB entries, and should thus be taken as the most probable within the context of plant N-glycosylation knowledge. The A to E glycoforms of γ-conglutin correspond to well-known N-glycan structures often seen in plants [28,37,39]. D and E are “complex-type” plant N-glycans, which differ from their mammalian counterparts for 1) the presence of core α1-3 fucose and core xylose β1-2-linked to the bisecting mannose, and 2) the lack of sialic acid and core α1-6 fucose. B and C, the most abundant N-glycans found in γ-conglutin, belong instead to the so-called “paucimannosidic-type” N-glycans, which are truncated variants of the “complex-type”. B and C are prototypical plant glycans, known as MUXF3 and MMXF3, that are well characterized as the major N-glycans in the model glycoproteins bromelain [40] and horseradish peroxidase [41], respectively.

To further substantiate the identity of the A to E glycoforms of Pept_127-165_ and Pept_111-165_, we verified the correspondence of the experimental and theoretical isotopic envelopes. Figure S3 shows two examples of the perfect matches obtained.

### Structural analysis of N-glycoforms by LC–MSn

To support the identity of the major A to E glycoforms, we analyzed shorter V8/trypsin glycopeptides by combined data-dependent LC–MS2 and targeted LC–MSn analysis. The shortest predicted peptides encompassing the two potential N-glycosylation sequons were STTSRPGCHN _131_N_132_TCGLISSNPVTQE (Pept_122-145_) and PGCHN _131_N_132_TCGLISSNPVTQE (Pept_127-145_).

#### Data dependent LC–MS2

The correct isotopic envelope of the MH3+ ions of the A to E glycoforms of both Pept122-145 and Pept127-145 appeared in the full MS spectra (not shown) within 1 ppm of the following theoretical monoisotopic mass of the MH3+ ions: 1165.170, 1209.184, 1263.202, 1330.895 and 1398.588 m/z for Pept122-145, and 987.750, 1031.764, 1085.782, 1153.475 and 1221.168 m/z for Pept127-145. The glycoform pattern of Pept122-145 and Pept127-145 was identical to that of Pept127-165 and Pept111-165, (i.e. B=C>D>E>A, Figure 2 and Table S2). As expected, the close elution of the A to E glycoforms was noted for each peptide (around 24.5 and 23.4 min respectively for Pept122-145 and Pept127-145). There was a few seconds difference in the retention time for the different glycoforms, with shorter glycoforms eluting later (Figure S4 for Pept122-145). The abundance of all Pept122-145 glycoforms was sufficient to trigger the acquisition of a single MS2 spectrum. Manual inspection of these spectra gave initial evidence in line with the saccharide composition of the hypothesized A to E glycoforms (Table 1). The sequence of the saccharide and peptide components of the A to E glycoforms of Pept122-145 was then confirmed by targeted MSn, as described below. 

#### Targeted LC–MSn

**Figure 4:**
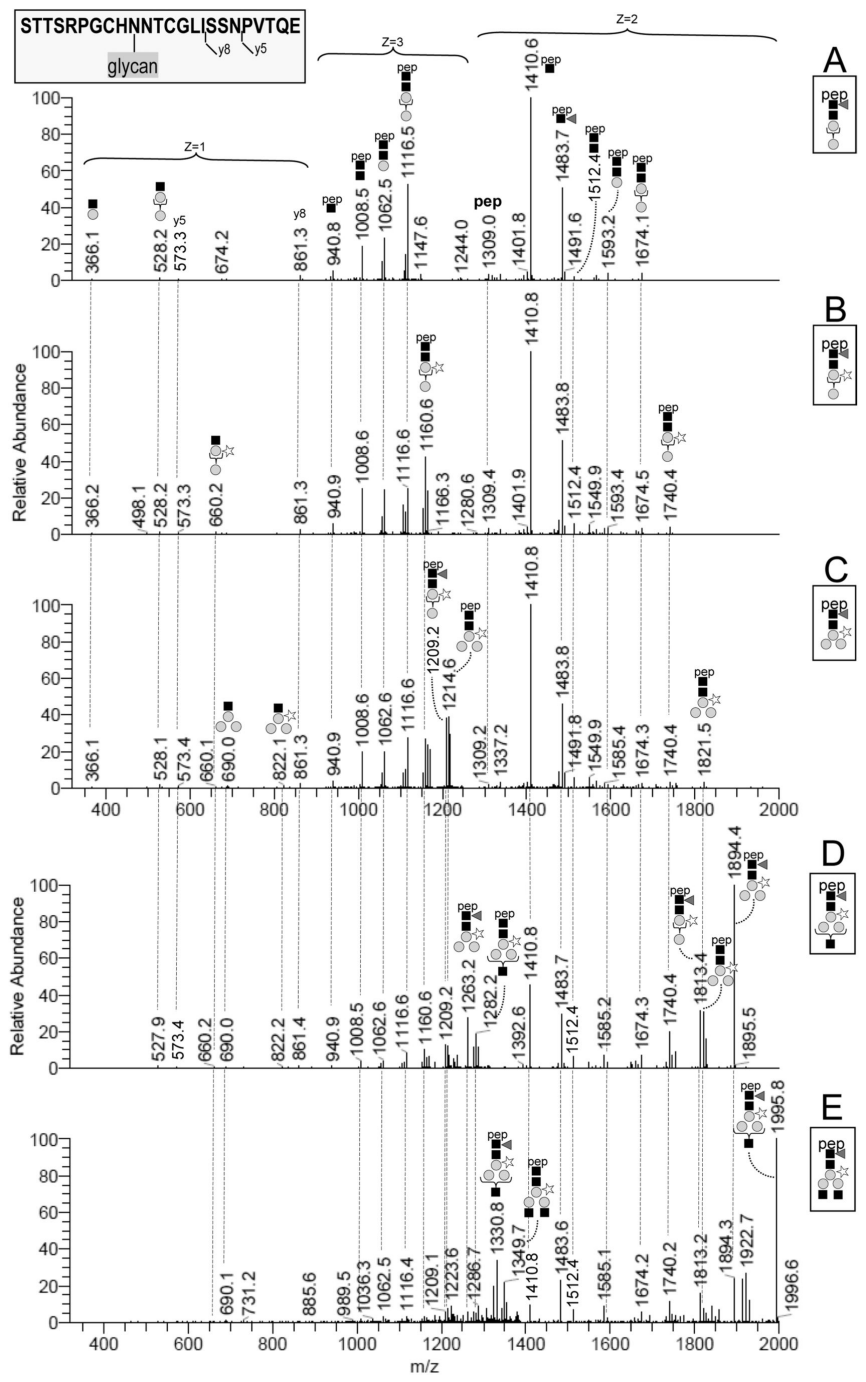
Annotated MS2 mass spectra of the A to E glycoforms of Pept_122-145_. The MS2 mass spectra were obtained by fragmenting MH^3+^ ions at 1165, 1209, 1263, 1331 and 1399 m/z (A to E glycoforms respectively). The dashed lines connect fragment ions with identical m/z values across the MS2 spectra. The fragments are annotated at their first appearance only (top to bottom), following the Consortium for Functional Glycomics Symbol Nomenclature. Braces within the glycan structure refer to possible alternative linkages (see Table S1).

The V8/trypsin digest was repeatedly analyzed by LC–MSn, each time targeting a different glycoform of Pept_122-145_. The instrument was set to repeat the following cycle during the LC run: 1) survey scan by Orbitrap (400-2000 m/z); 2) MS2 scan targeting for CID fragmentation the triply-charged ion of one selected glycoform; 3) MS3 scan targeting the MS2-product ion at m/z 1411 (z=2), a fragment corresponding to the intact peptide carrying a single HexNAc residue [42] (hereafter termed Pept+HexNAc), which is common to the five Pept_122-145_ glycoforms (Figure 4). The MS3 spectrum of the Pept+HexNAc ion was used to confirm the sequence of the peptide component. The MS2 spectra of the triply protonated A to E glycoforms of Pep_122-145_ (Figure 4) have in common several product ions and fragmentation patterns, in line with 1) the structural similarities of their saccharide component, and 2) the identity of the peptide element. An expected general characteristic of these spectra is the prevalent fragmentation of the glycan moiety. We observed the following main fragmentations: 1) the preferential cleavage of chitobiose (HexNAc–HexNAc) in the A to C glycoforms (without antennae), with production of a major Y_1_ fragment corresponding to Pept+HexNAc (m/z 1411 (z=2) and m/z 941 (z=3)); 2) the loss of a terminal non-reducing HexNAc residue from the intact glycopeptide ion in the glycoforms D and E; 3) the loss of core dHex (fucose) from the intact glycopeptide; 4) the sequential cleavage of all glycosidic bonds within the glycan, leading to ladders of abundant Y ions (Figure 4). In the low mass range, the MS2 spectra also showed oxonium ions that are diagnostic for N-glycopeptides (m/z 366=Hex–HexNAc, 528=Hex–Hex–HexNAc, 660=Hex–PentHex–HexNAc, 690=Hex_2_-Hex–HexNAc, 822=Hex_2_-PentHex–HexNAc) [43]. For all five glycoforms we also observed an abundant doubly charged fragment ion (m/z 1484) corresponding to Pept+dHexHexNAc, which unequivocally proves that the fucose residue is attached to the core GlcNAc [44]. Other interpretation details are reported in Figure 4.

**Figure 5:**
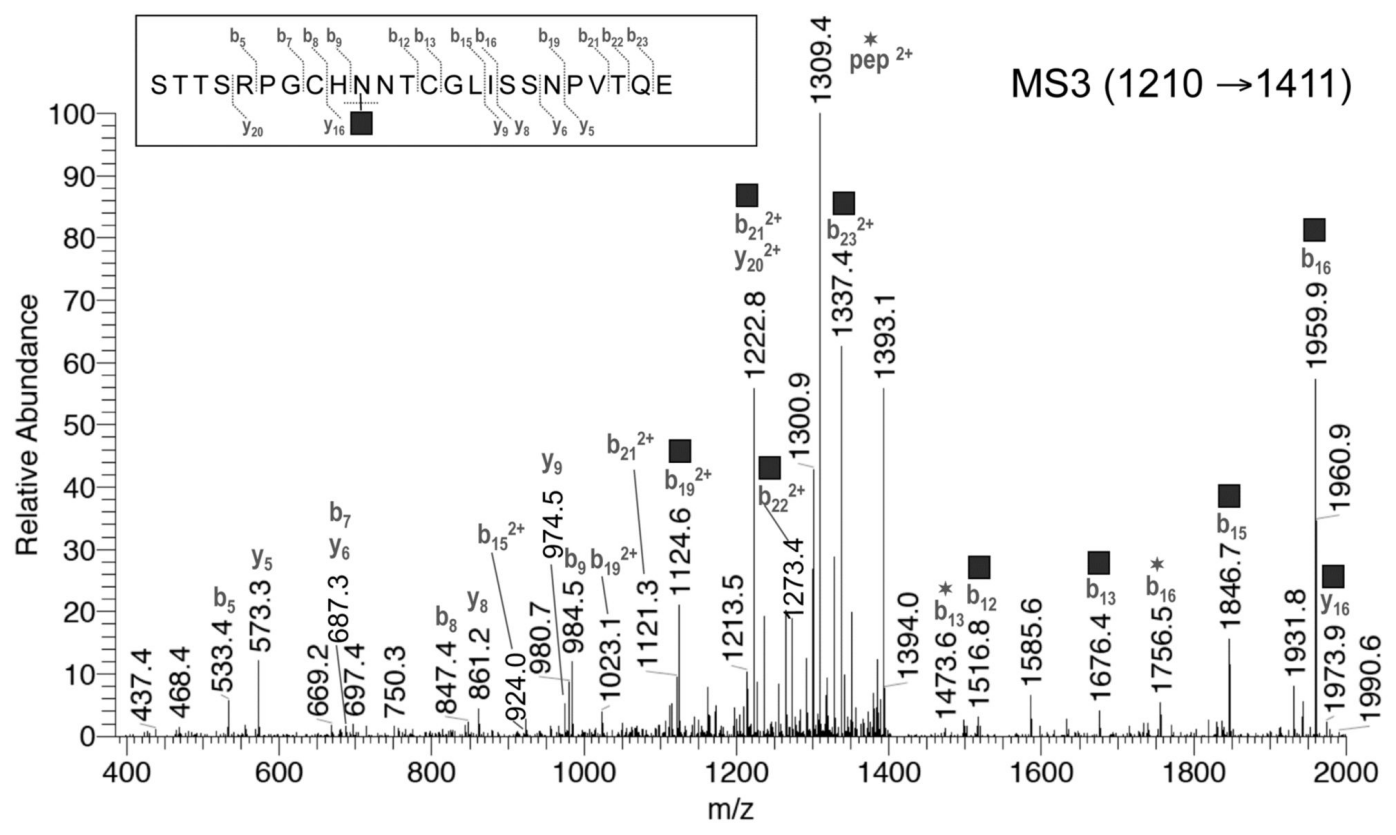
Representative annotated MS3 spectrum of the Pept+HexNAc product ion (m/z 1411, z=2). The Pept+HexNAc product ion derives from the MS2 fragmentation of the MH^3+^ ion (m/z 1210) of Pept_127-145_ glycoform B. The corresponding MS3 spectra for the other glycoforms were identical. The fragment ions with a *square* symbol are those with the HexNAc residue still in place, while those with a *star* have undergone the neutral loss of the HexNAc residue. HexNAc was annotated here at N_131_ based on evidence not related to this MS3 spectrum (see main text). Annotated fragments are within 0.6 Da from the theoretical value.

Identical MS3 spectra were obtained from the different glycoforms of Pep_122-145_ by fragmenting their common MS2 product ion Pept+HexNAc (m/z 1411, z=2). The MS3 fragmentation pattern clearly indicated that the peptide moiety is indeed STTSRPGCHNNTCGLISSNPVTQE. Figure 5 shows a representative annotated MS3 spectrum of the Pept+HexNAc product ion derived from the B glycoform (m/z 1210→1411). The main fragment ions are y- and b-type. The peptide fragments encompassing the two potential N-glycosylation sites (see next section) were mainly present with the HexNAc residue still in place. HexNAc (203 Da) was also lost from the intact Pept+HexNAc ion, as proved by the abundant doubly-charged fragment ion at m/z 1309.4. The MS3 spectra of Pept+HexNAc did not allow us to establish which of the adjacent N_131_ and N_132_ (positions 10 and 11 in Pep_122-145_) carries the N-glycosylation. However, we indicate N_131_ as the glycosylation site in Figures 4 and 5, having unequivocally clarified this by a different approach (see below).

#### Minor B′ to E′ glycoforms

A first compositional evidence of the minor variants of the B to E glycoforms lacking fucose (named here B′ to E′) was obtained for both tryptic peptides Pept_127-165_ and Pept_111-165_ (Figure 2). The data-dependent LC–MS2 analysis of the V8/trypsin digest of γ-conglutin then confirmed that the B′ to E′ glycoforms are indeed the fucose-free variants of the B to E glycoforms. All the fragments annotated in Figure 4 for the B to E glycoforms of Pept_122-145_ were in fact present in the MS2 spectra of the corresponding B′ to E′ glycoforms, with the exception of those containing fucose. The diagnostic ion (Pept+dHexHexNAc, m/z 1484), which supports the core position of fucose, was abundant in all the B to E glycoform MS2 spectra (Figure 4), but absent for all the B′ to E′ glycoforms. The MS2 spectra of the D′ and E′ glycoforms contained additional fragment ions clearly deriving from the fragmentation of another perfectly isobaric precursor. This pair of “contaminating” compounds were identified as an overalkylated form of the C and D glycoforms of Pept_122-145_ that carry one carbamidomethyl group in excess (+57.02 Da) [45]. A small satellite series (+57.02 Da) of the A to E glycoforms, representing overalkylated counterparts, had been observed in the MS spectra of the samples alkylated in solution. These satellite series were not seen in in-gel alkylated samples. We were aware that the B′ to E′ fucose-free series could derive from the in-source loss of fucose from the B to E glycoforms, but the LC–MS extracted ion chromatograms of the monoisotopic MH+ ions (±2 ppm) indicated that the fucosylated and non-fucosylated glycoforms did not exactly co-elute, thus proving that the fucose-free glycoforms do exist in the native protein.

### Selective N-deglycosylation

To obtain supporting evidence of the core α1-3 fucose in the A to E glycoforms, we used PNGase F (which cannot remove N-glycans that carry α1-3-linked core fucose) in parallel with PNGase A (which can remove all N-glycans). The A to E glycoforms of Pept_127-165_ and Pept_111-165_ remained substantially unchanged after PNGase F, but were both completely deglycosylated by PNGase A (Figure S5). The B′ to E′ fucose-free glycoforms of Pept_127-165_ and Pept_111-165_ instead disappeared after PNGase F, as expected (data not shown). The MS spectra of the putative deglycosylated Pept_111-165_ showed multicharged MH^+^ ions (z=4-7) that corresponded, after deconvolution and deisotoping, to a monocharged monoisotopic ion at 5954.675m/z. This indicates a +0.984 Da mass shift from the theoretical value of the non-glycosylated peptide (5953.698 m/z), supporting the actual N-deamidation, and thus N-deglycosylation, of Pept_111-165_ after PNGase A treatment. The same +0.984 Da mass shift was observed for PNGase A-treated Pept_127-165_.

### N-glycosylation site assignment

Two partially “overlapping” sequons are present in the γ-conglutin sequence (Asn_131_-Asn-Thr and Asn_132_-Thr-Cys [23]). The lack of peptide bond cleavage between the two adjacent asparagines meant that the MS2 spectra of the intact glycopeptides could not reveal which Asn carried the N-glycan. For unequivocal assignment of the glycosylation site, we therefore relied on the MS2 spectra of PGNase A-deglycosylated trypsin/V8 peptides. The deamidation of Asn caused by deglycosylation was easily detected as a +0.98 Da mass shift on Asn_131_ of Pept_127-145_ by MS2 analysis (Figure S6). Our results therefore clearly show that the microheterogeneous N-glycan in γ-conglutin is bound to Asn_131_.

### In-depth protein sequence coverage

An additional finding of this study is the extensive sequence coverage of γ-conglutin subunits provided by the global LC–MS2 analysis of the in-gel tryptic peptides. While the N-terminal sequence of the two *Lupinus* *albus* γ-conglutin subunits has been documented earlier by Duranti’s group [46], available MS-based proteomic data regarding γ-conglutin still leave large sections of the sequence uncovered [21,47]. Our present high-quality mass spectral data permitted high-confidence peptide identification with extensive protein sequence coverage (89%, Figure S7, and Table S4) confirming – with the exception of a few short amino acid stretches – the deduced amino acid sequence of γ-conglutin (UniProt Q9FSH9; NCBI gi|11191819). Using the “semi-trypsin” enzyme rule, i.e. a single trypsin-specific cleavage (C-term to K/R, but not before P) in the Mascot MS/MS search, we identified with high confidence: (a) a majority of strictly-tryptic peptides, (b) the N- and C-terminal semi-tryptic peptides of the two subunits, and (c) two peptides (Pept_111-126_ and Pept_127-165_) derived from the non-canonical “trypsin/P” cleavage (C-term to K/R, even before P) at R_126_–P_127_ (Figure S2). The latter finding is in line with recent studies [48,49] showing that [K/R].P type peptides can actually be cleaved by trypsin, more frequently if they “contain small amino acids as glycine, alanine, and serine” at both P2 and P2′ as it occurs in our sequence (…SR_126_–P_127_G…).

### Size and N-terminal sequence variants of the small subunit

The in-depth γ-conglutin sequence coverage helped explain the heterogeneity of the small subunit. While the C-terminus described by Duranti’s group [46] was confirmed by the unique C-terminal peptide (SCSNLFDLNNP_452_) identified here, we found – in addition to the most abundant N-terminal peptide S_301_YHESSEIGGAMITTTNPYTVLR that confirms the N-terminal sequence described earlier [22] – two additional N-terminal tryptic peptides, S_299_SSYHESSEIGGAMITTTNPYTVLR and S_297_SSSSYHESSEIGGAMITTTNPYTVLR. The existence of three major N-terminus variants in the native protein small subunit (Ser_301_, Ser_299_ and Ser_297_) with one, three, or five N-terminal serines implies that the small subunit variants had theoretical monoisotopic molecular masses of 16407.21, 16581.28 and 16755.34 Da, respectively. We confirmed this by measuring the accurate molecular mass of the intact small subunit variants with direct-infusion Orbitrap MS (Figure S8). The sequence of the N-terminus variants of this subunit could derive either from the “imprecise” S–S cleavage of the pro-polypeptide (at S_296_–S_297_, S_298_–S_299_, or S_300_–S_301_) or from successive steps of N-terminal proteolytical trimming of serines from the largest subunit variant having S_297_ as N-terminus.

## Conclusions

We characterized the N-glycosylation profile of γ-conglutin, and also defined the major N-terminal sequence variants of the heterogeneous small subunit.

By providing a general view of the structure and relative abundance of γ-conglutin glycoforms, we observed the prevalence of two “paucimannosidic-type” glycoforms (B and C: Man_2_(Xyl) (Fuc) GlcNAc_2_ and Man_3_(Xyl) (Fuc) GlcNAc_2_) and two less abundant “complex-type” N-glycans (D and E: GlcNAcMan_3_(Xyl) (Fuc) GlcNAc_2_ and GlcNAc _2_Man_3_(Xyl) (Fuc) GlcNAc_2_) [28]. We did not find significant proportions of other more complex, common plant N-glycans, e.g. the “high-mannose type”, or the “complex type” carrying the Lewis a (Le^a^) epitope [28].

N-glycans of the “paucimannosidic-type”, typical of vacuolar and seed storage glycoproteins, are truncated forms of “complex-type” N-glycans that lack terminal non-reducing GlcNAc [27,28]. The D to A glycoforms of γ-conglutin – carrying “paucimannosidic-type” N-glycans of decreasing complexity – likely results from the post-Golgi stepwise trimming of terminal monosaccharide residues from “complex-type” N-glycans matured in the Golgi compartment (i.e. the glycoform E, or larger Le^a^-containing “complex-type” N-glycans) [28,50].

The four most abundant N-glycans (B to E) alternatively attached to γ-conglutin carry two independent glyco-epitopes (core β1,2-xylose and core α1,3-fucose) that are widespread in plants but absent in humans [28,51]. Glycoproteins with these glyco-epitopes (known as Cross-reactive Carbohydrate Determinants, CCDs) [51], can elicit the production of antibodies in humans that, being specific for the carbohydrate target but not for the carrier proteins, can easily cross-react in *in vitro* allergy tests with non-homologous (but CCD-carrying) glycoproteins [27,51,52]. The actual contribution of CCDs to the allergenic potential of glycoproteins is an intricate and still controversial matter, and the clinical relevance of CCDs is highly debated [27,51,52,53]. Our identification of common CCDs in the major glycoforms of γ-conglutin could help in re-interpreting some of the conflicting data on the allergenic potential of this interesting bioactive glycoprotein.

## Supporting Information

Protocol S1 **Details are given for the following procedures: 1) Purification of γ-conglutin; 2) Proteolytic digestion; 3) N-deglycosylation; 4) Data analysis.**
(DOCX)

Table S1 **Mass list of the deconvoluted spectrum shown in Figure 1.**
(XLSX)

Table S2 **Relative abundance of the main series of the γ-conglutin glycoforms (A to E) based on the intensity of the deisotoped molecular ion in the deconvoluted Orbitrap MS spectra.**
(DOCX)

Table S3 **Linkage details of the plant N-glycan GlycoSuiteDB entries that univocally match the predicted composition of γ-conglutin A to E glycoforms.**
(DOC)

Table S4 **MASCOT peptide identification details.**
(XLS)

Figure S1 **SDS-PAGE analysis of purified γ-conglutin under non-reducing and reducing conditions.**
(TIF)

Figure S2 **Identification of non-glycosylated Pept_127-165_ by LC–MS2 and Mascot.** The identification was obtained analyzing the tryptic peptide/N-glycopeptide mixture obtained from in-gel digested γ-conglutin.
(TIF)

Figure S3 **Comparison of experimental and theoretical isotope envelopes.** Representative Orbitrap MS spectra (top panels) and simulated (bottom panels) isotope envelopes are shown for MH^5+^ of Pept_127-165_ glycoform C (chemical formula, with C-carbamidomethylation, +5H: C214H352N55O94S2) and MH^7+^ of Pept_111-165_ glycoform B (chemical formula, with C-carbamidomethylation, +7H: C281H457N80O118S4).
(TIF)

Figure S4 **Reverse-phase LC–MS analysis of A to E glycoforms of Pept_122-145_.** Extracted ion chromatograms (theoretical m/z value ± 5 ppm) are shown for the third isotopomer of the MH3+ ions. The signal intensity was normalized to the most abundant glycoform, to highlight the approximate relative abundance of the five glycoforms (B=C>D>E>A). The structures shown in the insets represent the N-glycans first hypothesized by MS and GlycoMod, then confirmed by targeted MSn experiments (see also Figure 2 in the main text).
(TIF)

Figure S5 **Effect of PNGase A on the A to E glycoforms, with Pept_111-165_ shown as an example.** LC–MS extracted ion chromatograms are shown for the A-E glycoforms and the N-deglycosylated (N-deamidated) Pept_111-165_. The MH^6+^ ions were extracted using the theoretical m/z values of the fifth isotopomer (± 2.5 ppm). The A-E glycoforms of Pept_111-165_, clearly visible in the control sample (left panel), completely disappeared after PNGase A-treatment (right panel), while the expected large amount of the previously absent N-deamidated (i.e. N-deglycosylated) Pept_111-165_ appeared at the expected retention time, i.e. right after the smallest (A) glycoform. All glycoform traces are normalized to the highest intensity (3.5E6) for easier comparison. The intensity of the N-deamidated peptide peak is 2.2E7.
(TIF)

Figure S6 **Mascot identification of the N-deamidation site within Pept_127-145_, which encompasses the two potential N-glycosylation sequons (N_131_NT and N_132_TC).** The MS2 fragments show that N-deamidation occurred at N_131_ and not N_132_, since there is a +0.98 Da mass shift in all the y and b fragments that include N_131_ (y_15_ to y_18_, and b_5_ to b_18_), but not in the fragments including N_132_ but not N_131_ (y_3_ to y_14_).
(TIF)

Figure S7 **In-depth sequence coverage of γ-conglutin.** The deduced sequence of γ-conglutin chain (positions 34-452, without the signal peptide 1-33) is shown in black, while the sequence covered by peptide identification by Mascot with “semitrypsin” as enzyme is in blue or red. The N-terminal region of γ-conglutin corresponds to the large subunit (blue), and the C-terminal region to the small subunit (red). Dots indicate the short unidentified amino acid sequences. Detailed Mascot results are given for all the identified peptides in Table S2. The three underlined red serine (**S**) positions indicate the three alternative N-termini of the small subunit that were first suggested by peptide identification, then confirmed by the accurate mass of the intact small subunit variants analyzed by direct-infusion Orbitrap MS (Figure S8).
(TIF)

Figure S8 **Enlarged view of the MS spectrum of γ-conglutin small subunit variants.** The averaged spectrum showed – after charge-deconvolution and deisotoping – three abundant ions with monoisotopic neutral mass values (16407.16, 16581.26, and 16755.33 Da) that are within 3 ppm from the theoretical values of the three sequence variants of the small subunit.
(TIF)
