# Supplementary material for: In-Depth Glycoproteomic Characterization of γ-Conglutin by High-Resolution Accurate Mass Spectrometry
Source: PLoS One. 2013 Sep 12;8(9):e73906. doi: 10.1371/journal.pone.0073906 (PMC3771881; doi:10.1371/journal.pone.0073906)
Supplement: Protocol S1 — Details are given for the following procedures: 1) Purification of γ-conglutin; 2) Proteolytic digestion; 3) N-deglycosylation; 4) Data analysis. (DOCX) [file pone.0073906.s001.docx]

PROTOCOL S1

**Purification of γ-conglutin.** *Lupinus albus* γ-conglutin was kindly supplied by Professor M. Duranti (University of Milan, Italy).

γ-Conglutin was extracted and purified as follows. Defatted lupin flour was suspended in 50 mM sodium phosphate buffer pH 7.5, containing 0.5 M NaCl (1:20, w/v). After stirring for 4 h at 4°C, the suspension was centrifuged at 10,000 x g for 30 min at 4°C. The supernatant was then desalted on a Sephadex G-50 column, equilibrated with 50 mM sodium phosphate buffer (pH 7.5), and loaded onto a Whatman DE 52 DEAE-cellulose column equilibrated in the same buffer. The unbound fraction containing γ-conglutin was adjusted to pH 4.5 with acetic acid, loaded on a Whatman CM-cellulose column equilibrated in 50 mM sodium acetate buffer (pH 4.5), and then eluted by addition of 0.35 M NaCl in the same buffer. A further purification step was performed by insulin-agarose immobilized affinity chromatography, with γ-conglutin solution loaded on the column equilibrated in 25 mM Tris-HCl (pH 7.2), and eluted by addition of 0.2 M NaCl to the loading buffer. In the following purification step by metal affinity chromatographic separation, the protein solution was loaded onto a nickel column (NiNTA-Agarose, QIAGEN, Milan, Italy) equilibrated in 50 mM Tris-HCl (pH 7.4), containing 0.5 M NaCl. The protein bound to the matrix was eluted with 50 mM sodium acetate (pH 4.5) containing 0.5 M NaCl. The purified protein was dialyzed at 4°C with frequent changes of milliQ water, and freeze-dried.

**Proteolytic digestion.** In-gel trypsin digestion (with reduction and carbamidomethylation) was done on gel-separated γ-conglutin subunits or monomer bands according to Schiarea et al. LC–MS analysis was done on 2-µl aliquots of the digests (25 µl).In solution V8/trypsin digestion was done by (a) reduction (10 mM DTT, 56°C, 1 h) and carbamidomethylation (5 mg/ml iodoacetamide, T amb, 30 min); (b) incubation (overnight, 37°C) with V8 (Roche, Mannheim, Germany) in 0.1 M phosphate buffer pH 7.5 (enzyme/substrate mass ratio, 1:20); (c) dilution 1:1 (v/v) in 0.1 M ammonium bicarbonate (pH 8) and digestion overnight at 37°C with trypsin (Roche) (enzyme/substrate mass ratio 1:20). LC–MS analysis was done on 1-µl aliquots of the digests (diluted to 0.5 µg/µl with water).

**N-deglycosylation**. Dried digests of the large subunit band were treated with two N-glycosidase enzymes of different specificity, i.e. PNGase A and F, while dried V8/trypsin digests were deglycosylated by PNGase A only. Tryptic digests (3 µg) were resuspended in 50 µl phosphate buffer (0.1 M, pH 7.5), incubated overnight (37°C) with 2.5 units of PNGase F (Roche). PNGase A (Europa Bioproducts Ltd, Cambridge, UK) was used as follows: 25 µU of enzyme were incubated overnight (37°C) with dried trypsin digests (3 µg) or V8/trypsin digests (30 µg) resuspended in 10 or 25 µl citrate-phosphate buffer (50 mM, pH 5), respectively. Samples were analyzed by LC-MS (2µl injected).

**Data analysis.**

*Automated peptide identification.* The Mascot search engine was used to identify non-glycosylated and deglycosylated peptides in the various digests. MS2 spectra were exported into .dta files by Bioworks browser 3.3.1 (Thermo Scientific). MS2 spectra originating from the same precursor ion were grouped together (2 ppm tolerance, intensity >100 counts and >10 ions, automatic charge state assignment). Dta files were merged, and submitted as an .mgf file to Mascot search engine (in-house version 2.2.07, Matrix Science, Boston, MA). The MS2 ion search was done against the NCBInr database 20120531 (18280215 sequences; 6265275233 residues), with the following parameters: taxonomy, “Viridiplantae” (1078290 sequences); no restriction on molecular weight or isoelectric point; fixed modification, carbamidomethylation of cysteine; variable modification (where appropriate): oxidation of methionine (for in-gel digest) and deamidation of asparagine (for PNGase-treated samples); one missed cleaveage allowed; experimental mass values, monoisotopic; peptide mass tolerance, ±5 ppm; MS2 mass tolerance, ±1 Da; peptide charge, 2+, 3+, 4+; enzyme, “trypsin”, “semitrypsin”, or “none” (when trypsin and V8 were both used).
