# Supplementary material for: In-Depth Glycoproteomic Characterization of γ-Conglutin by High-Resolution Accurate Mass Spectrometry
Source: PLoS One. 2013 Sep 12;8(9):e73906. doi: 10.1371/journal.pone.0073906 (PMC3771881; doi:10.1371/journal.pone.0073906)
Supplement: Table S2 — Relative abundance of the main series of the γ-conglutin glycoforms (A to E) based on the intensity of the deisotoped molecular ion in the deconvoluted Orbitrap MS spectra. (DOCX) [file pone.0073906.s003.docx]

**Table S2**. Relative abundance of the main series of the γ-conglutin glycoforms (A to E) based on the intensity of the deisotoped molecular ion in the deconvoluted Orbitrap MS spectra.

|  |  | **Glycoform relative abundance (%)** | | |  |
| --- | --- | --- | --- | --- | --- |
| **N-glycoform** | **N-glycan structure** | **Pept_127-165_** | **Pept_111-165_** | **Pept_122-145_** | **mean±SD** |
| A | MUF^3^/MF^3^ | 4.4 | 4.6 | 4.5 | 4.5±0.1 |
| B | MUXF^3^/UMXF^3^ | 33.3 | 33.6 | 31.5 | 32.8±1.1 |
| C | MMXF^3^ | 33.9 | 33.4 | 33.5 | 33.6±0.3 |
| D | MGnXF^3^/GnMXF^3^ | 18.7 | 18.8 | 20.2 | 19.2±0.8 |
| E | GnGnXF^3^ | 9.6 | 9.6 | 10.3 | 9.8±0.4 |
| All |  | 100 | 100 | 100 |  |

Note: The A to E glycoforms of Pept_127-145_ had an identical abundance pattern, as judged from theMH^3+^ ions (too low signals for MS spectra deconvolution).
