# Supplementary material for: In-Depth Glycoproteomic Characterization of γ-Conglutin by High-Resolution Accurate Mass Spectrometry
Source: PLoS One. 2013 Sep 12;8(9):e73906. doi: 10.1371/journal.pone.0073906 (PMC3771881; doi:10.1371/journal.pone.0073906)
Supplement: Table S3 — Linkage details of the plant N-glycan GlycoSuiteDB entries that univocally match the predicted composition of γ-conglutin A to E glycoforms. (DOC) [file pone.0073906.s004.doc]

**Table S3.** Linkage details of the plant N-glycan GlycoSuiteDB entries that univocally match the predicted composition of γ-conglutin A to E glycoforms.

|  | **N-Glycan**  **composition** | **Univocally matching, specific N-glycan structures**  (GlycoSuiteDB, plant organisms only) | **ProGlycAn**  **nomenclature^a^** |
| --- | --- | --- | --- |
| **A** | Hex_2_HexNAc_2_dH | isomer I Man(α1-6)Man(β1-4)GlcNAc(β1-4)[Fuc(α1-3)]GlcNAc  isomer II Man(α1-3)Man(β1-4)GlcNAc(β1-4)[Fuc(α1-3)]GlcNAc | MUF^3^  UMF^3^ |
| **B** | Hex_2_HexNAc_2_dHex_1_Pent_1_ | isomer I Man(α1-6)[Xyl(β1-2)]Man(β1-4)GlcNAc(β1-4)[Fuc(α1-3)]GlcNAc  isomer II Man (α1-3)[Xyl(β1-2)]Man(β1-4)GlcNAc(β1-4)[Fuc(α1-3)]GlcNAc | MUXF^3^  UMXF^3^ |
| **C** | Hex_3_HexNAc_2_dHex_1_Pent_1_ | Man(α1-3)[Man(α1-6)][Xyl(β1-2)]Man(β1-4)GlcNAc(β1-4)[Fuc (α1-3)]GlcNAc | MMXF^3^ |
| **D** | Hex_3_HexNAc_3_dHex_1_Pent_1_ | isomer I GlcNAc(β1-2)Man(α1-6)[Man(α1-3)][Xyl(β1-2)]Man(β1-4)GlcNAc(β1-4)[Fuc(α1-3)]GlcNAc  isomer II GlcNAc(β1-2)Man(α1-3)[Man(α1-6)][Xyl(β1-2)]Man(β1-4)GlcNAc(β1-4)[Fuc(α1-3)]GlcNAc | MGnXF^3^  GnMXF^3^ |
| **E** | Hex_3_HexNAc_4_dHex_1_Pent_1_ | GlcNAc(β1-2)Man(α1-6)[GlcNAc(β1-2)Man(α1-3)][Xyl(β1-2)]Man(β1-4)GlcNAc(β1-4)[Fuc(α1-3)]GlcNAc | GnGnXF^3^ |

^a^Abbreviation system for N-glycan structures (<http://www.proglycan.com/index.php?page=pga_nomenclature>)

.
