# Supplementary figures and images for: In-Depth Glycoproteomic Characterization of γ-Conglutin by High-Resolution Accurate Mass Spectrometry
Source: PLoS One. 2013 Sep 12;8(9):e73906. doi: 10.1371/journal.pone.0073906 (PMC3771881; doi:10.1371/journal.pone.0073906)

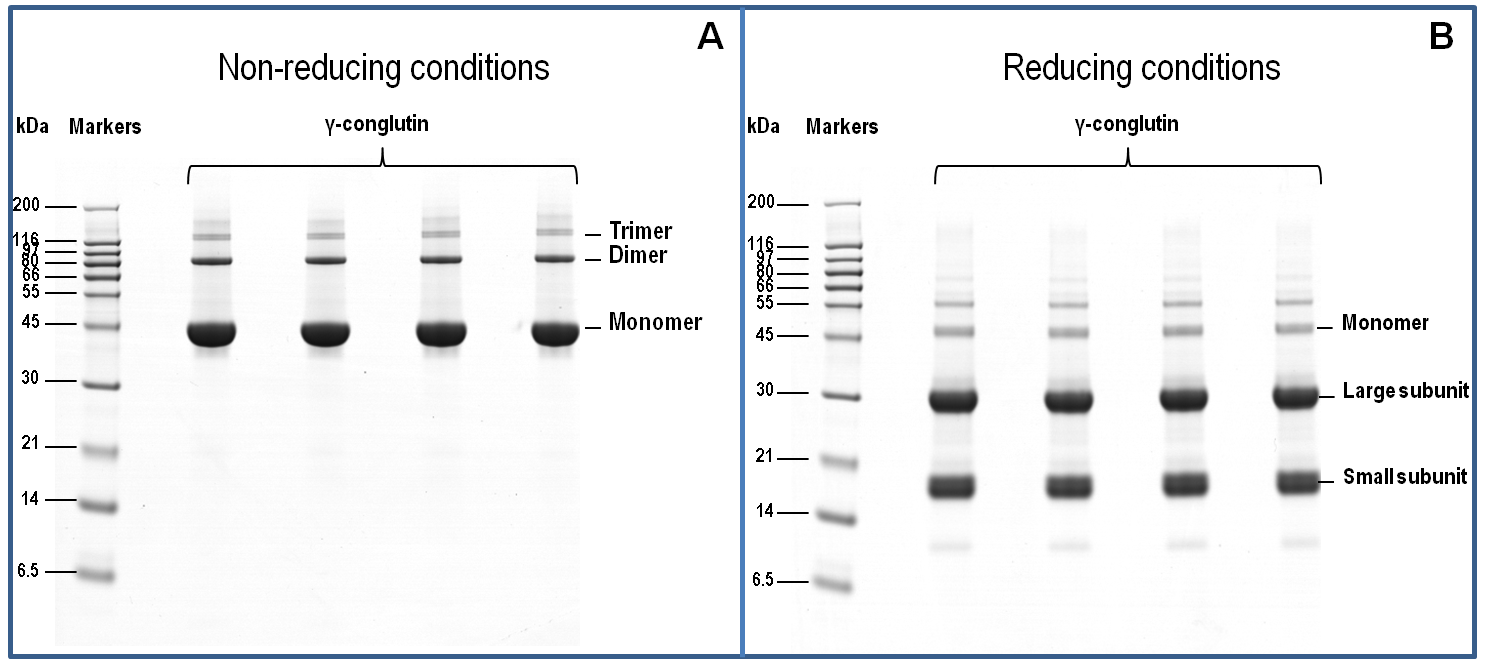

Supplement: Figure S1 — SDS-PAGE analysis of purified γ-conglutin under non-reducing and reducing conditions. (TIF) [file pone.0073906.s006.tif]

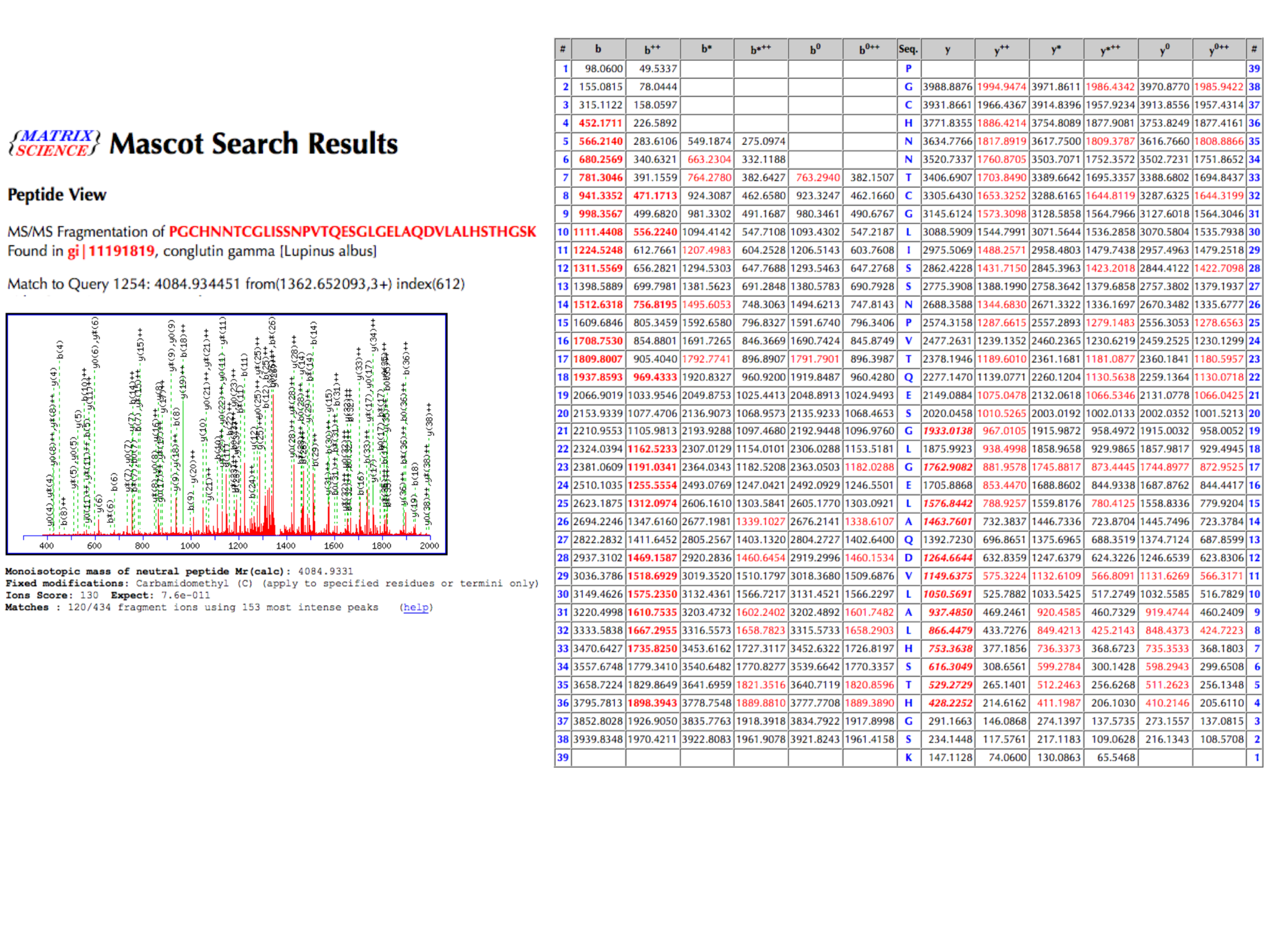

Supplement: Figure S2 — Identification of non-glycosylated Pept127-165 by LC–MS2 and Mascot. The identification was obtained analyzing the tryptic peptide/N-glycopeptide mixture obtained from in-gel digested γ-conglutin. (TIF) [file pone.0073906.s007.tif]

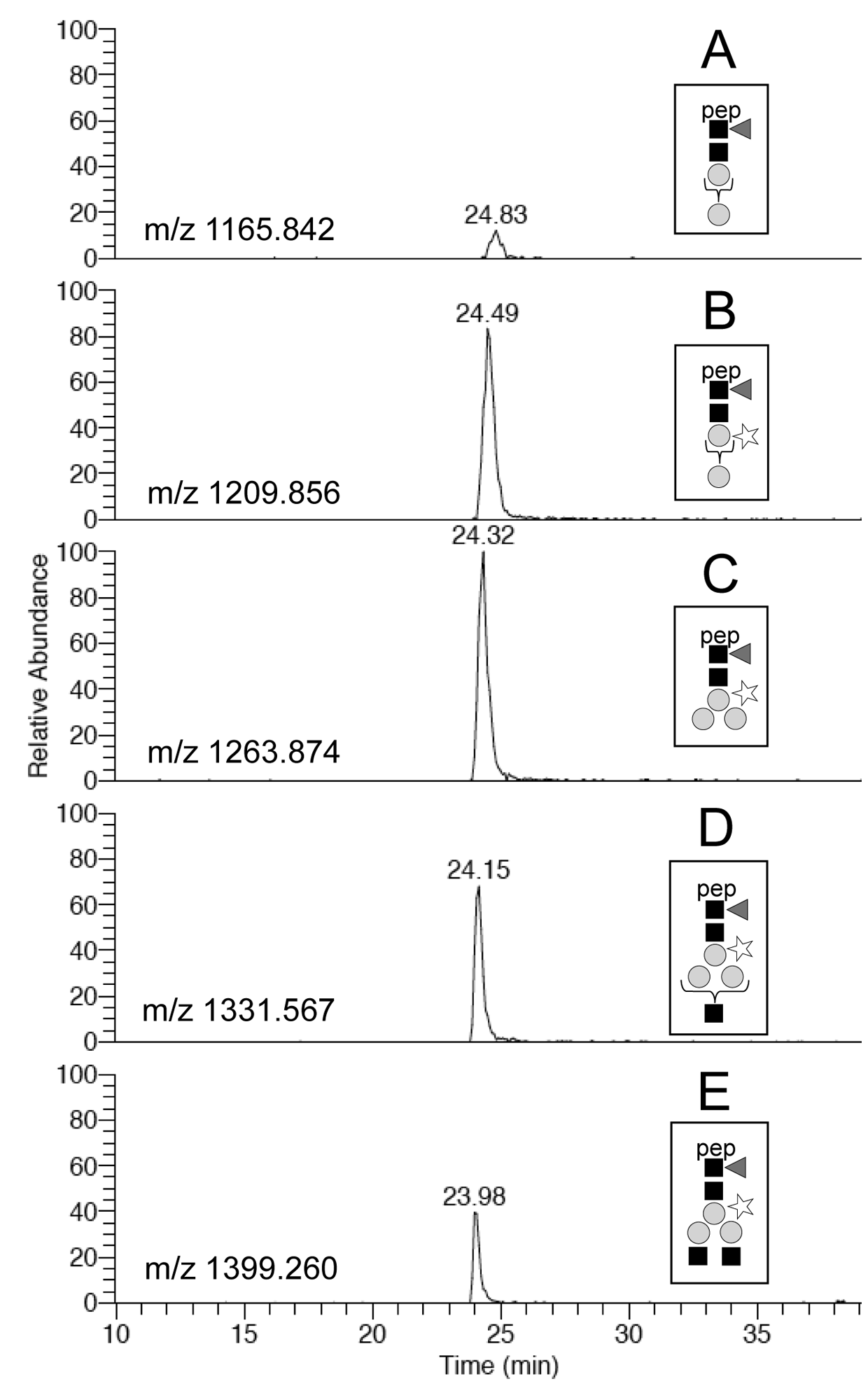

Supplement: Figure S4 — Reverse-phase LC–MS analysis of A to E glycoforms of Pept122-145. Extracted ion chromatograms (theoretical m/z value ± 5 ppm) are shown for the third isotopomer of the MH3+ ions. The signal intensity was normalized to the most abundant glycoform, to highlight the approximate relative abundance of the five glycoforms (B=C>D>E>A). The structures shown in the insets represent the N-glycans first hypothesized by MS and GlycoMod, then confirmed by targeted MSn experiments (see also Figure 2 in the main text). (TIF) [file pone.0073906.s009.tif]

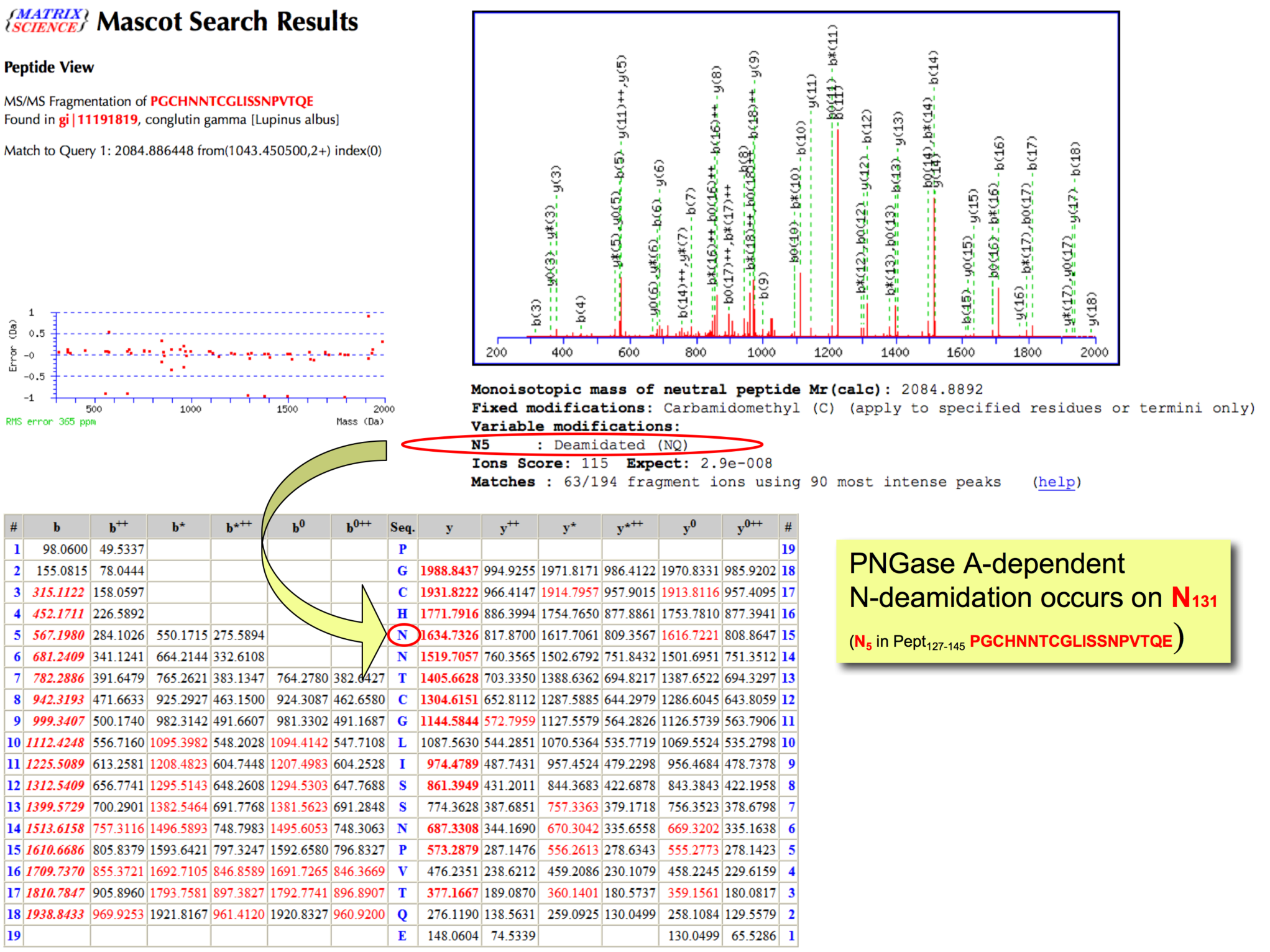

Supplement: Figure S6 — Mascot identification of the N-deamidation site within Pept127-145, which encompasses the two potential N-glycosylation sequons (N131NT and N132TC). The MS2 fragments show that N-deamidation occurred at N131 and not N132, since there is a +0.98 Da mass shift in all the y and b fragments that include N131 (y15 to y18, and b5 to b18), but not in the fragments including N132 but not N131 (y3 to y14). (TIF) [file pone.0073906.s011.tif]

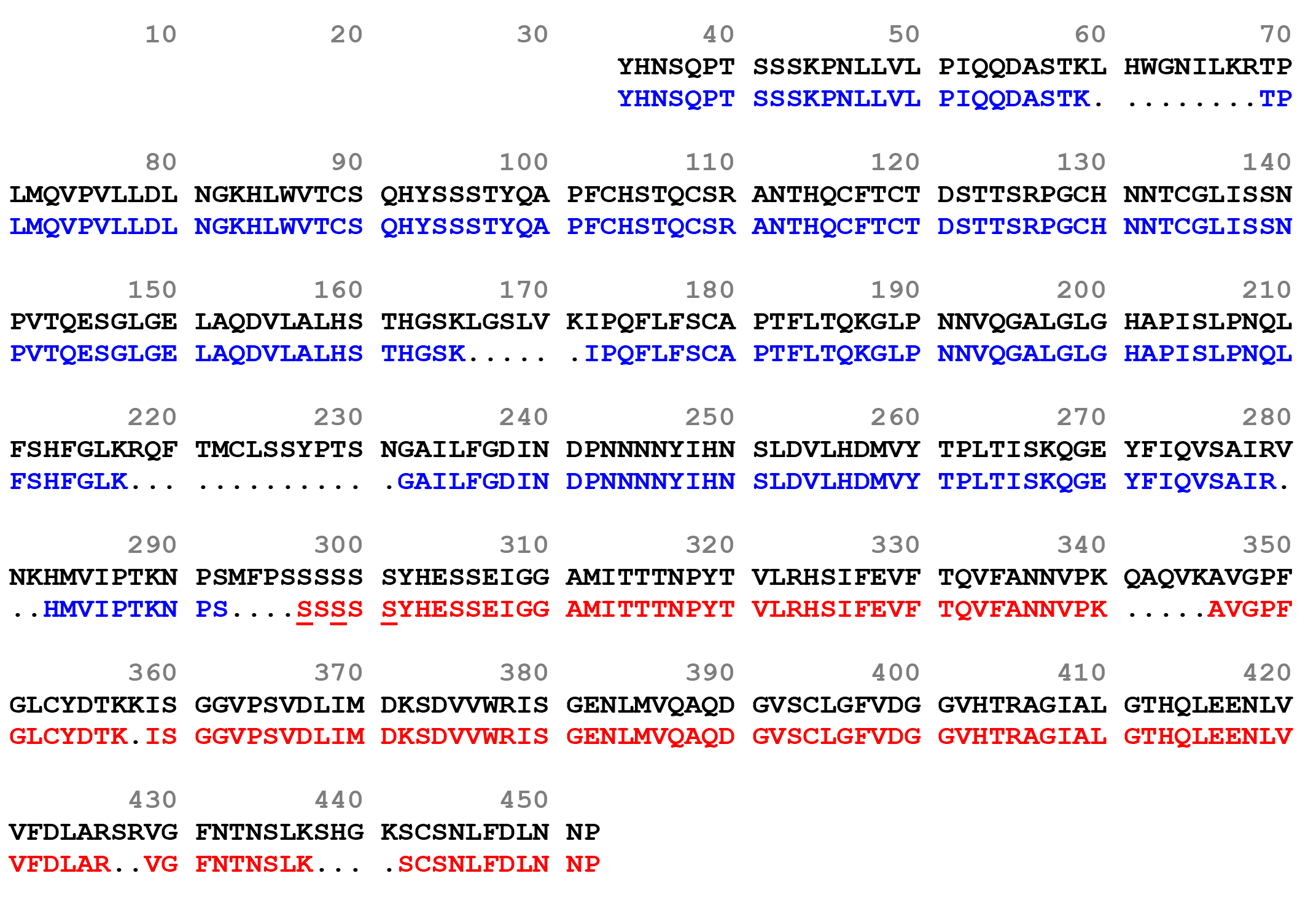

Supplement: Figure S7 — In-depth sequence coverage of γ-conglutin. The deduced sequence of γ-conglutin chain (positions 34-452, without the signal peptide 1-33) is shown in black, while the sequence covered by peptide identification by Mascot with “semitrypsin” as enzyme is in blue or red. The N-terminal region of γ-conglutin corresponds to the large subunit (blue), and the C-terminal region to the small subunit (red). Dots indicate the short unidentified amino acid sequences. Detailed Mascot results are given for all the identified peptides in Table S2. The three underlined red serine (S) positions indicate the three alternative N-termini of the small subunit that were first suggested by peptide identification, then confirmed by the accurate mass of the intact small subunit variants analyzed by direct-infusion Orbitrap MS (Figure S8). (TIF) [file pone.0073906.s012.tif]
